# Royal Free Hospital-Nutritional Prioritizing Tool improves the prediction of malnutrition risk outcomes in liver cirrhosis patients compared with Nutritional Risk Screening 2002

**DOI:** 10.1017/S0007114520002366

**Published:** 2020-12-28

**Authors:** Yuchao Wu, Yage Zhu, Yali Feng, Ruojing Wang, Naijuan Yao, Mengmeng Zhang, Xiaohui Liu, Huan Liu, Lei Shi, Li Zhu, Nan Yang, Hongmei Chen, Jinfeng Liu, Yingren Zhao, Yuan Yang

**Affiliations:** 1 Department of Infectious Diseases and Hepatopathy, The First Affiliated Hospital of Xi’an Jiaotong University, Xi’an, Shannxi Province, 710061, People’s Republic of China; 2 The First Department of Orthopedics, The Second Affiliated Hospital of Xi’an Jiaotong University, Xi’an, Shannxi Province, 710004, People’s Republic of China; 3 Center for Infectious Diseases, West China Hospital, Sichuan University, Chengdu, Sichuan Province, 610041, People’s Republic of China

**Keywords:** Malnutrition, Cirrhosis, Nutritional Risk Screening 2002, Royal Free Hospital-Nutritional Prioritizing Tool, Prognostic value, LDUST, Liver Disease Undernutrition Screening Tool, MAC, mid-arm circumference, MELD, Model for End-stage Liver Disease, MUST, Malnutrition Universal Screening Tool, NRS-2002, Nutritional Risk Screening 2002, RFH-GA, Royal Free Hospital-Global Assessment, RFH-NPT, Royal Free Hospital-Nutritional Prioritizing Tool, TSF, triceps skinfold thickness

## Abstract

The European Society for Clinical Nutrition and Metabolism (ESPEN) guidelines recommend the Royal Free Hospital-Nutritional Prioritizing Tool (RFH-NPT) to identify malnutrition risk in patients with liver disease. However, little is known about the application of the RFH-NPT to screen for the risk of malnutrition in China, where patients primarily suffer from hepatitis virus-related cirrhosis. A total of 155 cirrhosis patients without liver cancer or uncontrolled co-morbid illness were enrolled in this prospective study. We administered the Nutritional Risk Screening 2002 (NRS-2002), RFH-NPT, Malnutrition Universal Screening Tool (MUST) and Liver Disease Undernutrition Screening Tool (LDUST) to the patients within 24 h after admission and performed follow-up observations for 1·5 years. The RFH-NPT and NRS-2002 had higher sensitivities (64·8 and 52·4 %) and specificities (60 and 70 %) than the other tools with regard to screening for malnutrition risk in cirrhotic patients. The prevalence of nutritional risk was higher under the use of the RFH-NPT against the NRS-2002 (63 *v*. 51 %). The RFH-NPT tended more easily to detect malnutrition risk in patients with advanced Child–Pugh classes (B and C) and lower Model for End-stage Liver Disease scores (<15) compared with NRS-2002. RFH-NPT score was an independent predictive factor for mortality. Patients identified as being at high malnutrition risk with the RFH-NPT had a higher mortality rate than those at low risk; the same result was not obtained with the NRS-2002. Therefore, we suggest that using the RFH-NPT improves the ability of clinicians to predict malnutrition risk in patients with cirrhosis primarily caused by hepatitis virus infection at an earlier stage.

Malnutrition is a very common and serious complication of cirrhosis, with a prevalence ranging from 60 to 85 %^(1–4)^. The onset and/or severity of malnutrition proceeds from a compromised nutritional state to an obvious loss of weight and finally to a lean body mass. It is not only associated with the progression of liver dysfunction^(5)^ but is also related to complications of liver cirrhosis, such as infections, hepatic encephalopathy and ascites^(6,7)^. Early nutritional intervention is essential to reduce the length of hospital stay and healthcare-associated costs, improve quality of life and decrease the mortality rate^(8,9)^. However, nutritional intervention is usually delayed owing to the failure to assess the risk of malnutrition and the differences in the accuracy of screening tools^(10)^.

Nutritional risk is defined as the ‘chances of a better or worse outcome from disease or surgery according to actual or potential nutritional and metabolic status’^(11)^. Several nutrition screening tools have been developed to predict the potential or existing risk of disease-related malnutrition. In particular, the Nutritional Risk Screening 2002 (NRS-2002) and Royal Free Hospital-Nutritional Prioritizing Tool (RFH-NPT) are validated screening tools for hospitalised patients that are recommended by the European Society of Parenteral Enteral Nutrition guidelines^(12)^. The RFH-NPT was first developed in a multicentre trial in the UK to detect nutritional status in patients with chronic liver disease^(13)^. It is easily applied in the clinical setting, which enables even non-specialist staff to efficiently utilise the tool, saving time. Patients are separated into low-, medium- or high-risk categories according to five measurements, including BMI, unplanned weight loss, dietary intake, the severity of hepatitis and interference with food intake by current complications (e.g. ascites, general fluid overload). The NRS-2002 is another simple tool primarily based on the indications for nutritional support in 2002^(14)^. It combines several variables, including the percentage of weight loss, BMI, a reduction in food intake and the presence of disease and its severity^(14,15)^. A comparison suggested that the RFH-NPT was more sensitive than the NRS-2002 for the identification of liver patients at risk for malnutrition^(16,17)^. However, the studies regarding the use of the RFH-NPT to assess the risk of malnutrition in patients with liver disease were mainly performed in Europe^(16,17)^, where alcoholic liver disease is the primary aetiology of liver cirrhosis and there is a higher prevalence of malnutrition. In contrast, most cases of liver cirrhosis are due to viral hepatitis in China, rendering the evaluation of alcohol consumption in the RFH-NPT irrelevant in Chinese populations. Therefore, we investigated whether the assessment of the risk of malnutrition by the RFH-NPT was still superior to assessment with the NRS-2002 in China by comparing them with the results obtained with the Royal Free Hospital-Global Assessment (RFH-GA).

## Patients and methods

### Patients

We conducted a prospective study with eligible adult patients who were seen in the Department of Infectious Diseases and Hepatopathy at the First Affiliated Hospital of Xi’an Jiaotong University between October 2015 and May 2017. We screened 258 patients who had been diagnosed with cirrhosis based on clinical, biochemical, histological, radiological (ultrasound or computed tomography showing a lobulated liver or/and unequivocal signs of portal hypertension) and/or elastographic (defined as liver stiffness >14 kPa) criteria and advanced disease. Patients were subsequently excluded if they also had liver cancer or if they had any uncontrolled co-morbid illness, such as uncontrolled diabetes mellitus, tuberculosis, AIDS, chronic renal failure, muscle disease, rheumatological disease, disease of the digestive tract, parasitic disease or active drug abuse. Patients with uncontrolled joint disease and neuropathy were also excluded. Finally, a total of 155 cirrhosis patients were enrolled in the present day and were followed up until the date of death or for 1·5 years. After receiving informed consent from each patient, blood samples, which were used for biochemistry assessments, and anthropometric parameters were collected. The nutritional risk screening assessments were administered according to the respective protocols within 24 h after the admission of each patient to the department^(4)^. This project was approved by the ethics committee at Xi’an Jiaotong University.

### Nutrition status assessment

In liver cirrhosis patients, nutritional status can be assessed using the RFH-GA^(18)^. The RFH-GA incorporates both subjective and objective variables to assess nutrition, including BMI, mid-arm muscle circumference and dietary intake. In the case of fluid retention, BMI needs to be corrected for the patient’s dry weight, commonly estimated by the postparacentesis body weight or the weight recorded before fluid retention if available, or by subtracting a percentage of the weight based upon the severity of the ascites (mild 5 %; moderate 10 % and severe 15 %), with an additional 5 % subtracted if bilateral pedal oedema is present, as described in several studies. Then, the dry-weight BMI is calculated by dividing the patient’s estimated dry weight (kg) by the square of the patient’s height (m). The triceps skinfold thickness (TSF, mm) and mid-arm circumference (MAC, cm) were measured using Holtain/Tanner-Whitehouse skinfold calipers. These two measurements are used to calculate the mid-arm muscle circumference: MAMC (cm) = MAC (cm) – (3·14 × TSF(cm)). Measures of the mid-arm muscle circumference below the 5th percentile are indicative of a risk of malnutrition.

With acknowledgement of the European Association for the Study of the Liver (EASL) as an optimal one for dietary assessment in patients with cirrhosis^(19)^, the random 2-d 24-h dietary recall is an easy and rapid method to conduct^(20,21)^. As described previously^(20,21)^, the food and beverage consumption of the participants was collected over 2 d (one weekday and one weekend day) by our experienced dietitian. The individual intakes of energy and nutrients relating to metabolic diseases on each day were calculated according to the China Food Composition^(20)^. The dietary intake was estimated and categorised as adequate if it met the requirements calculated by Schofield’s modification of the Harris–Benedict equation, inadequate if it failed to meet the estimated requirements but exceeded 2092 kJ/d (500 kcal/d) and negligible if it provided <2092 kJ/d (<500 kcal/d)^(18)^.

### Nutritional risk screening tools

NRS-2002: The NRS-2002 is a nutrition screening tool recommended by the European Society for Clinical Nutrition and Metabolism guidelines^(14)^. It includes three components: the nutritional score (BMI, weight loss and dietary intake), the disease severity score and the age score (age > 70 years)^(15)^. Patients are classified as having no or low risk when they have a total score <3 or as having a moderate or high risk when they have a total score ≥3.

RFH-NPT: As described previously, the RFH-NPT is a novel nutrition screening tool developed in the UK^(13)^. It includes three major steps: (1) those who have alcoholic hepatitis or are undergoing tube feeding are immediately evaluated as high risk without proceeding to the next step; (2) those who do not have alcoholic hepatitis and are not undergoing tube feeding are assessed for fluid overload and its impact on food intake and weight loss and (3) those who do not have fluid overload are assessed for nutritional status (BMI, unplanned weight loss and daily dietary intake). Patients are stratified as being at low risk if they have a score of 0, moderate risk if they have a score of 1 and high risk if they have a score of 2–7.

Malnutrition Universal Screening (MUST): The MUST includes three categories: current BMI, unintentional weight loss and the presence of any acute disease that could compromise nutritional intake for >5 d.

Liver Disease Undernutrition Screening Tool (LDUST): The LDUST assesses the six factors that were identified as having the strongest associations with malnutrition in patients with a chronic disease^(22)^. It consists of six questions addressing these factors, namely, nutrient intake, weight loss, loss of subcutaneous fat, loss of muscle mass, fluid accumulation and a decline in functional status. The three potential patient responses are labelled column A, column B and column C, indicating no signs of undernutrition, ‘mild to moderate’ undernutrition and ‘moderate to severe’ undernutrition.

### Medical assessment

Anthropometric measurements, such as BMI (kg/m^2^), MAC (cm) and TSF (mm), were obtained by specialist staff in our department. The measurements of BMI, MAC and TSF were described previously^(2)^.

Laboratory parameters associated with malnutrition, such as the total serum protein level (g/l), albumin level (g/l) and total lymphocyte count, were obtained from routine clinical laboratory measurements (RCLM) in the Department of Clinical Laboratory of First Affiliated Hospital of Xi’an Jiaotong University.

Based on biochemical data collected from medical records, the Model for End-stage Liver Disease (MELD) and Child–Pugh scores were calculated to estimate disease severity.

### Sample size estimation

The sample size was estimated based on the malnutrition rates in patients with cirrhosis of 44·6 % assessed by the NRS-2002 and 50·7 % assessed by the RFH-NPT^(16)^. The reported sensitivity and specificity of nutritional risk screening by the NRS-2002 vary widely, with values of 50–86 and 21–93 %, respectively^(23)^. The calculated minimum sample size was 96, with a sensitivity and specificity of 50 %, an *α* of 0·05 and a power of 85 %^(24)^.

### Statistical analyses

Statistical analyses were performed using SPSS 23.0. The continuous variables are described as mean values and standard deviations or as medians with ranges. The differences between means were analysed using independent Student’s *t* tests. Nominal variables are described as numbers or percentages, and their differences were analysed with Pearson’s *χ*^2^ test, Fisher’s exact test or the McNemar test. Observations were collected until a mean of 1·5 years from admission. The sensitivity and specificity were calculated for the various screening tools compared with the RFH-GA. Receiver operating characteristic curve analysis was performed using the RFH-GA as the reference. Cox regression analysis, Kaplan–Meier analysis and *χ*^2^ tests were conducted for the prediction and comparison of survival in patients with NRS-2002 scores <3 *v*. those with NRS-2002 scores ≥3 and those with RFH-NPT scores <2 *v*. those with RFH-NPT scores ≥2 at admission. Survival curves were generated using GraphPad Prism 5. Differences were considered significant at *P* < 0·1 for the receiver operating characteristic curve analysis and *P* < 0·05 for the other analyses.

## Results

### General characteristics of patients

The main patient characteristics are presented in Table 1. A total of 155 patients were ultimately enrolled (females 61, 39·4 %; males 94, 60·6 %) in the analysis. The mean age was 49·40 (sd 5·30) (median 56, range 33–61) years. The majority of patients (139/155, 90 %) had hepatitis virus-related cirrhosis; 1 % (2/155) of the patients were affected by alcohol-related cirrhosis and the remaining 9 % (14/155) of the patients had other aetiologies. Among those with hepatitis virus-related cirrhosis, 59·4 % (92/155) of the patients were infected with hepatitis B virus (HBV), and 30·3 % (47/155) were infected with hepatitis C virus (HCV). All hepatitis virus-infected patients received antiviral treatment in our study (data not shown). The mean patient MELD score was 6·80 (sd 3·02). Fifty-nine (38 %) patients had Child–Pugh class C cirrhosis. A total of 31·6 % (49/155) of the patients were diagnosed with compensated cirrhosis, and 68·4 % (106/155) had decompensated cirrhosis. A total of 9·7 % (15/155) and 46·5 % (72/155) of the patients presented with hepatic encephalopathy and ascites, respectively. All patients with encephalopathy received treatment with branched-chain amino acids, and all patients with ascites received treatment with diuretics (data not shown). The overall median BMI, MAC and TSF were 26·17 (20·42–27·41) kg/m^2^, 29·0 (25·5–32·0) cm and 23·0 (9·0–36·0) mm, respectively. Twenty-five (16·1 %) patients died during follow-up, and 16 (10·3 %) were lost to follow-up due to incorrect contact information. In addition, no patients died from drinking alcohol.

**Table 1.** Patients’ overall characteristics (Mean values and standard deviations; median values and ranges; numbers and percentages)

Index	Mean		sd
Age (years)	49·40		5·30
Sex			
Female			
*n*		94	
%		60·6	
Male			
*n*		61	
%		36·7	
BMI (kg/m^2^)	24·68		1·31
Dry weight BMI (kg/m^2^)	23·05		3·71
MAC (cm)	28·5		1·22
MAMC (cm)	20·1		2·95
TSF (mm)	22·60		4·88
Total serum protein (g/l)	79·72		2·65
Albumin (g/l)	37·16		2·41
Prealbumin (mg/l)	132·34		46·25
Total bilirubin (µmol/l)			
Median		26·7	
Range		10·20–275·10	
Na (mmol/l)	140·92		0·37
SCr (µmol/l)	3·96		0·81
BUN (mmol/l)	5·73		3·99
Ferroprotein (ng/ml)			
Median		142·9	
Range		41·91–2149·00	
INR	1·25		0·09
PT (s)	15·54		0·82
PTA (%)	70·16		12·05
Total lymphocyte count (×10^9^/l)	1·27		0·16
Blood ammonia (µmol/l)	46·8		18·00
Child–Pugh score	7·40		0·93
MELD score	6·80		3·02
NRS-2002 score	2·60		0·678
RFH-NPT score	1·40		0·75
Cirrhosis			
Compensated			
*n*		49	
%		31·6	
Decompensated			
*n*		106	
%		68·4	
Aetiology of cirrhosis			
Alcoholism			
*n*		2	
%		1·3	
Chronic viral hepatitis			
*n*		139	
%		89·7	
Others			
*n*		14	
%		9·0	
Aetiology of cirrhosis			
Hepatitis B virus			
*n*		92	
%		59·4	
Hepatitis C virus			
*n*		47	
%		30·3	
Child–Pugh			
A			
*n*		44	
%		28·4	
B			
*n*		52	
%		33·5	
C			
*n*		59	
%		38·1	
MELD			
<15			
*n*		118	
%		76·1	
>15			
*n*		37	
%		23·9	
NRS 2002			
No to low risk			
*n*		76	
%		49·0	
No to high risk			
*n*		79	
%		51·0	
RFH-NPT			
Low risk			
*n*		57	
%		36·8	
Moderate to high risk			
*n*		98	
%		63·2	
Hepatic encephalopathy			
Absent			
*n*		140	
%		90·3	
Present			
*n*		15	
%		9·7	
Ascites			
Absent			
*n*		83	
%		53·5	
Present			
*n*		72	
%		46·5	
Deceased			
*n*		25	
%		16·1	
Living			
*n*		114	
%		73·5	
Lost to follow-up			
*n*		16	
%		10·3	

MAC, mid-arm circumference; MAMC, mid-arm muscle circumference; TSF, triceps skinfold thickness; SCr, serum creatinine; BUN, blood urea nitrogen; INR, international normalised ratio; PT, prothrombin time; PTA, plasma thromboplastin antecedent; MELD, Model for End-stage Liver Disease; NRS-2002, Nutritional Risk Screening 2002; RFH-NPT, Royal Free Hospital-Nutritional Prioritizing Tool; MELD, Model for End-stage Liver Disease.

The basic clinical parameters in patients with or without decompensated cirrhosis are shown in online Supplementary Table S1. The sex and age distributions did not differ between the groups with and without decompensated cirrhosis. The compensated and decompensated cirrhosis groups had no significant differences in BMI or the levels of serum creatinine, blood urea nitrogen and ferroprotein. The other remaining clinical parameters were significantly lower in the decompensated cirrhosis group than in the group without decompensated cirrhosis. Decompensated cirrhosis patients had relatively higher Child–Pugh class cirrhosis and MELD, NRS-2002 and RFH-NPT scores than compensated cirrhosis patients. Among the 106 decompensated cirrhosis patients, 14·2 % (15/106) and 70 % (71/106) presented with hepatic encephalopathy and ascites, respectively. In summary, patients diagnosed with decompensated cirrhosis had worse anthropometric measurements and laboratory parameters than patients with compensated cirrhosis.

### Effectiveness of nutrition risk screening tools compared with the Royal Free Hospital-Global Assessment

**Fig. 1.**
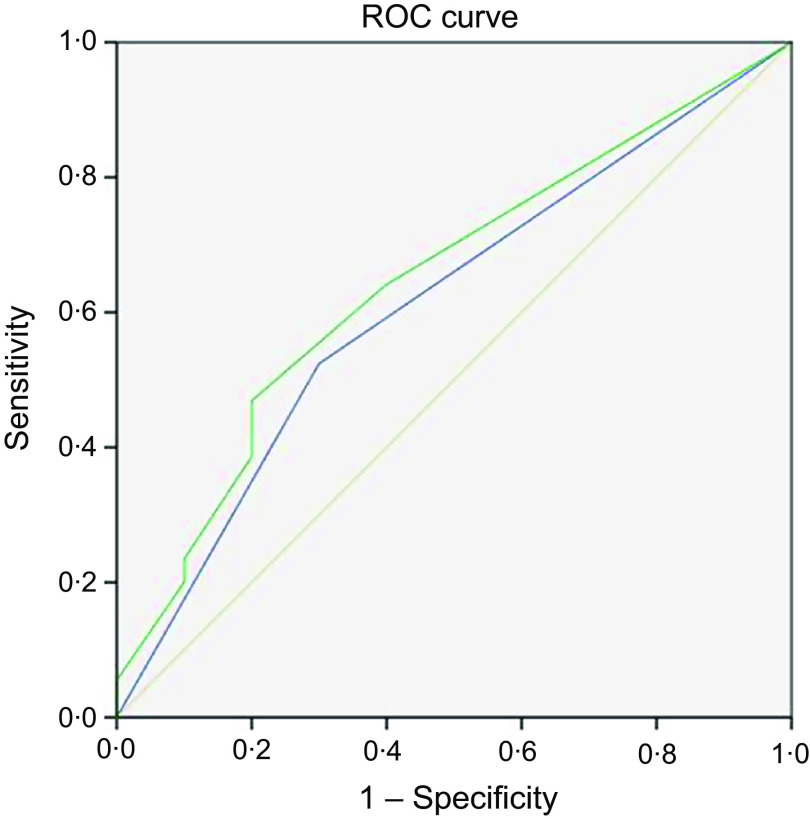
Receiver operating characteristic (ROC) curves of the screening tools for the prediction of nutritional risk with the Royal Free Hospital-Global Assessment (RFH-GA) as a reference. Diagonal segments are produced by sites. Source of the curve: 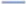, Nutritional Risk Screening 2002; 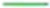, Royal Free Hospital-Nutritional Prioritizing Tool; 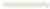, reference line.

The RFH-GA is a subjective index for nutritional assessment. To determine which screening tools accurately detected nutritional risk, we analysed the effectiveness of each tool by comparing its performance with that of the RFH-GA. The LDUST had the highest sensitivity and lowest specificity of the screening tools (69 and 10 %, respectively); the MUST had the lowest sensitivity (38·6 %) and the RFH-NPT had a higher sensitivity than the NRS-2002 (sensitivity 64·8 and 52·4 %, respectively) (Table 2). According to the receiver operating characteristic curve analysis (Fig. 1), the RFH-NPT and NRS-2002 were able to predict nutritional risk in patients with liver cirrhosis (AUC 0·647 and 0·612, respectively). We thus selected the RFH-NPT and NRS-2002 for more detailed analyses.

**Table 2.** Diagnostic value of the nutritional screening tools compared with the Royal Free Hospital-Global Assessment (RFH-GA)* (Percentages)

Screening tools	Malnutrition risk (%)	Sensitivity (%)	Specificity (%)	*P*
LDUST	70·3	69	10	0·082
RFH-NPT	63·2	64·8	60	0·085
MUST	38·1	38·6	70	0·091
NRS-2002	51·0	52·4	70	0·089

LDUST, Liver Disease Undernutrition Screening Tool; MUST, Malnutrition Universal Screening Tool; NRS-2002, Nutritional Risk Screening 2002.

* Malnutrition prevalence as assessed by the reference method (RFH-GA) was 69·5 %.

### Association of malnutrition risk identified by the NRS-2002 or the Royal Free Hospital-Nutritional Prioritizing Tool with poor clinical parameters

No statistically significant difference in sex distribution was shown between patients with low malnutrition risk and those with moderate to high risk (online Supplementary Table S2). The patients with low malnutrition risk according to the NRS-2002 were older than those in the moderate- and high-risk group, but there was no difference in the age distributions when the risk of malnutrition was assessed by the RFH-NPT. Compared with the low-risk group identified by the RFH-NPT, the levels of total serum protein and albumin and the total lymphocyte count were all significantly lower in the high-risk group; the same result was not obtained when the risk of malnutrition was assessed with the NRS-2002. Other clinical parameters were poor in patients with a moderate to high risk of malnutrition defined both by the NRS-2002 and RFH-NPT.

### Royal Free Hospital-Nutritional Prioritizing Tool was superior for the detection of the risk of malnutrition in patients with cirrhosis

To determine the advantages of one assessment over the other, we performed *χ*^2^ tests for detailed comparisons. A higher percentage of patients was identified as having moderate or high malnutrition risk scores by the RFH-NPT (98/155 or 63 %) than by the NRS-2002 (79/155 or 51 %; *P* = 0·001; Table 3). Patients with cirrhosis were stratified into compensated and decompensated groups, and nutritional risk was assessed in each group with the NRS-2002 and RFH-NPT. The same results were obtained with the NRS-2002 and RFH-NPT among compensated patients (11/49 or 22·4 % *v*. 11/49 or 22·4 %, *P* = 1; online Supplementary Table S3). However, the RFH-NPT identified more patients at risk for malnutrition in the decompensated group than the NRS-2002 (89/106 or 84 % *v*. 68/106 or 64 %, *P* < 0·001; online Supplementary Table S4).

**Table 3.** Nutrition assessment comparison between the Nutritional Risk Screening 2002 (NRS-2002) and Royal Free Hospital-Nutritional Prioritizing Tool (RFH-NPT) in all patients (Numbers)

NRS-2002	RFH-NPT		Total	*P*
	Low risk	High risk		
Low risk	51	25	76	
High risk	6	73	79	0·001
Total	57	98	155	

### Royal Free Hospital-Nutritional Prioritizing Tool identified the risk of malnutrition related to cirrhosis severity

We evaluated the relationship of the NRS-2002 and RFH-NPT assessments with the severity of cirrhosis as defined by the following: (1) Child–Pugh class, (2) MELD score, with or without, (3) hepatic encephalopathy and (4) ascites. First, ability of the RFH-NPT was not significantly different from that of the NRS-2002 with regard to identifying patients with Child–Pugh class A cirrhosis who were at moderate to high risk for malnutrition (Table 4, *P* = 1), although there was a non-significant difference between the two assessments in patients with Child–Pugh class B cirrhosis (Table 4, 34/52 or 65·4 % *v*. 27/52 or 51·9 %, *P* = 0·065, respectively). However, the RFH-NPT identified 22 % (13/59) more Child–Pugh class C patients who were at risk for malnutrition compared with the NRS-2002 (55/59 or 93·2 % *v*. 42/59 or 71·2 %, *P* = 0·002, Table 4). Second, the RFH-NPT, compared with the NRS-2002, was better able to detect patients at moderate and high risk for malnutrition (64/118 or 54·2 % *v*. 52/118 or 44·1 %, *P* = 0·012, Table 5) in the group of patients with MELD scores <15. For patients with MELD scores >15, there was no significant difference in the numbers of patients identified as being at risk for malnutrition by the RFH-NPT and the NRS-2002. Third, among patients with ascites, 27·8 % (20/72) were still categorised as having a low risk of malnutrition by the NRS-2002, but none was classified as having a low risk of malnutrition by the RFH-NPT (online Supplementary Table S5). Finally, we evaluated nutritional risk in patients with hepatic encephalopathy. The RFH-NPT was better able to identify potential malnutrition in patients without hepatic encephalopathy when compared with the NRS-2002 (84/140 or 60 % *v*. 66/140 or 47·1 %, *P* = 0·001, online Supplementary Table S6). No significant difference was found between the two tools in patients with hepatic encephalopathy (online Supplementary Table S6).

**Table 4.** Nutrition risk screening comparison between the Nutritional Risk Screening 2002 (NRS-2002) and Royal Free Hospital-Nutritional Prioritizing Tool (RFH-NPT) in patients with different Child–Pugh classes (Numbers)

Child–Pugh class	RFH-NPT	NRS-2002		Total	*P*
		Low risk	High risk		
A	Low risk	33	2	35	1
	High risk	1	8	9	
	Total	34	10	44	
B	Low risk	16	2	18	0·065
	High risk	9	25	34	
	Total	25	27	52	
C	Low risk	2	2	4	0·002
	High risk	15	40	55	
	Total	17	42	59	

**Table 5.** Nutrition risk screening comparison between the Nutritional Risk Screening 2002 (NRS-2002) and Royal Free Hospital-Nutritional Prioritizing Tool (RFH-NPT) based on Model for End-stage Liver Disease (MELD) scores (Numbers)

MELD score	RFH-NPT	NRS-2002		Total	*P*
		Low risk	High risk		
<15	Low risk	50	4	54	0·012
	High risk	16	48	64	
	Total	66	52	118	
≥15	Low risk	1	2	3	0·065
	High risk	9	25	34	
	Total	10	27	37	

### Royal Free Hospital-Nutritional Prioritizing Tool is a better predictor of survival

**Fig. 2.**
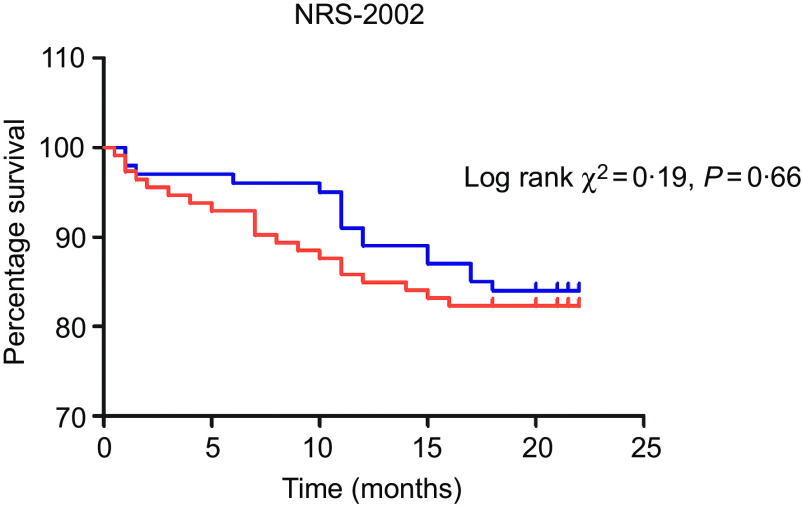
Kaplan–Meier curves for the patients categorised as having a low risk or a moderate to high risk of malnutrition by the Nutritional Risk Screening 2002 (NRS-2002). 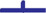, Low risk; 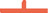, high risk.

**Fig. 3.**
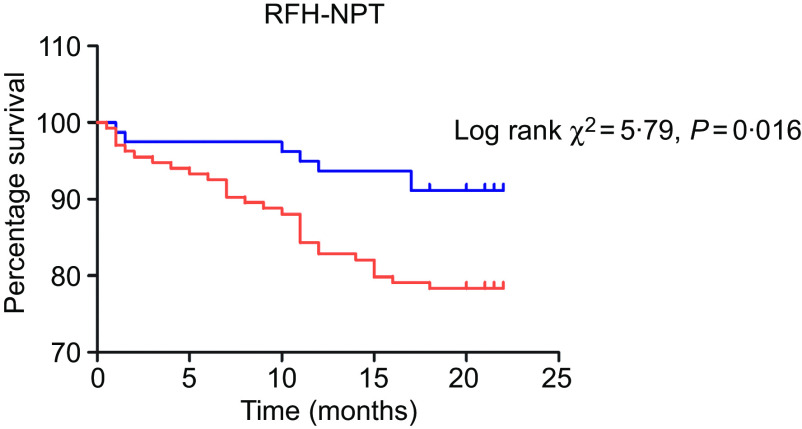
Kaplan–Meier curves for the patients categorised as having a low risk or a moderate to high risk of malnutrition by the Royal Free Hospital-Nutritional Prioritizing Tool (RFH-NPT). 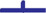, Low risk; 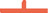, high risk.

Kaplan–Meier curves were used to estimate the prognostic value of each nutrition screening tool. The analysis was run from the day after hospital admission to the date of death or a median of 1·5 years. The moderate to high risk of malnutrition identified by the RFH-NPT was significantly associated with poor survival (Fig. 2, *P* = 0·019). Patients evaluated as having a moderate to high risk of malnutrition by the NRS-2002 did not differ in survival from those at low risk (Fig. 3, *P* = 0·50). Furthermore, we assessed the predictive power of the two tools for patient mortality in subgroups of patients with compensated and decompensated cirrhosis. No patients died in the compensated group in our study. However, the RFH-NPT was better able than the NRS-2002 to detect the risk of malnutrition in living patients with decompensated cirrhosis (55/70 or 78·6 % *v*. 45/70 or 64·3 %, *P* = 0·031, online Supplementary Table S8). Among the patients who died due to decompensation, 28 % (7/25) were misclassified as having a low risk of malnutrition by the NRS-2002 but were deemed to be at moderate or high risk of malnutrition by the RFH-NPT (21/25 or 84 % *v*. 14/25 or 56 %, *P* = 0·016; online Supplementary Table S8). Multivariate Cox regression analysis showed that the RFH-NPT but not the NRS-2002 was an independent factor predicting the survival of cirrhosis patients, with an OR of 2·041 (95 % CI 1·12, 3·73, *P* = 0·02) (Table 6).

**Table 6.** Multivariate Cox regression analysis of factors correlated with time to death (Odds ratios and 95 % confidence intervals)

Variables	OR	95 % CI	*P*
MAMC	1·057	0·70, 1·59	0·79
TSF	1·025	0·92, 1·14	0·656
Albumin	1·017	0·83, 1·25	0·874
Prealbumin	0·977	0·93, 1·03	0·364
Total bilirubin	1·02	1·00, 1·04	0·018
Na	1·00	0·84, 1·19	0·984
INR	0·01	0, 1100	0·257
PT (s)	1·257	0·525, 3·01	0·607
PTA (%)	0·85	0·59, 1·22	0·38
Total lymphocyte count (×10^9^/l)	0·05	0·01, 0·97	0·048
NRS-2002 score at inclusion	0·463	0·19, 1·15	0·097
RFH-NPT score at inclusion	2·041	1·12, 3·73	0·02

MAMC, mid-arm muscle circumference; TSF, triceps skinfold thickness; INR, international normalised ratio; PT, prothrombin time; PTA, plasma thromboplastin antecedent; NRS-2002, Nutritional Risk Screening 2002; RFH-NPT, Royal Free Hospital-Nutritional Prioritizing Tool.

## Discussion

The liver plays an important role in the metabolism of carbohydrates, proteins and fat^(25,26)^. Dysfunction of the liver contributes to malnutrition, which can develop occultly in the early stage of chronic liver disease and can ultimately lead to protein-energy malnutrition^(6,7)^. The identification of patients at risk of malnutrition in a hospital setting with an effective and simple nutrition risk screening tool is essential; such a tool would enable earlier nutrition assessment and more timely interventions, leading to reductions in morbidity and mortality in patients with cirrhosis^(6)^.

Previous European studies suggested that the RFH-NPT was more sensitive than the NRS-2002 for the assessment of the risk of malnutrition and the prediction of disease progression and outcomes in patients with chronic liver disease based on regression analysis^(11,12,16,17)^. Although the RFH-NPT is recommended as a screening tool for malnutrition in liver disease patients, there are few data from Asia. With a relatively large population of patients with liver disease in China, we directly compared the RFH-NPT and NRS-2002 with regard to the assessment of malnutrition in patients stratified by liver function, disease severity and survival status. In Europe, alcohol consumption is the primary cause of liver cirrhosis. A European study found that malnutrition is more prevalent in patients with alcoholic cirrhosis than in those with viral cirrhosis^(17)^. The RFH-NPT specially takes alcohol-related variables into consideration. Therefore, cirrhosis patients tend to be predicted to have a higher risk of malnutrition when the RFH-NPT is used than when general nutrition screening tools are used. However, many cases of liver cirrhosis in Asia are caused by viral hepatitis, which does not require the evaluation of alcohol consumption. We investigated whether the RFH-NPT is still more effective than other scores for nutrition screening in China. In addition, the NRS-2002 includes variables of disease severity and complications. The NRS-2002 is considered helpful for identifying malnourished liver cirrhosis patients with hepatocellular carcinoma^(12)^. Our study excluded factors such as hepatocellular carcinoma or uncontrolled co-morbid illness and made a direct and clear comparison of the scores in patients whose nutritional risk status was primarily affected by liver cirrhosis.

Our findings demonstrate that the RFH-NPT is more sensitive than the NRS-2002 for the identification of patients at risk for malnutrition in the early stage of liver disease. The overall prevalences of malnutrition obtained by the RFH-NPT and NRS-2002 were 63 % (98/155) and 51 % (79/155) in patients with cirrhosis, respectively. Our study showed a similar prevalence of malnutrition to those reported in other studies, which varied from 55 to 70 % as assessed by the RFH-NPT^(3)^ and from 31 to 45 % as assessed by the NRS-2002^(27,28)^.

Malnutrition is prevalent in all forms of liver disease, ranging from 20 % in compensated liver disease to >80 % in patients with decompensated liver disease^(29–31)^. The presented results suggested that the prevalence of malnutrition identified by the RFH-NPT varied from 22·4 % in patients with compensated cirrhosis to 84·0 % in patients with decompensated cirrhosis; the prevalences of malnutrition identified by the NRS-2002 were 22·4 and 64·2 % in patients with compensated and decompensated cirrhosis, respectively. The high level of agreement with regard to the prevalence of malnutrition in patients with compensated cirrhosis supports the validity of the RFH-NPT.

Malnutrition in patients with cirrhosis usually contributes to an increased rate of the development of ascites^(32)^. The NRS-2002, developed by Kondrup et al. and the European Society for Clinical Nutrition and Metabolism, was validated based on a meta-analysis of 128 trials^(15)^. Subsequent studies have suggested that the ability and sensitivity of the NRS-2002 with regard to the identification of patients at risk of malnutrition are highly variable among disease populations and age groups^(33)^. A high false-negative rate of 36–38 % with the NRS-2002 was detected in hospitalised patients^(34)^, indicating that more than one-third of patients at risk for malnutrition would be misclassified. As fluid collections (ascites, peripheral oedema) conceal weight loss occurring from muscle or fat loss, inaccurate weight and BMI measurements result in the underestimation of the risk of malnutrition at the first screening of patients with cirrhosis, especially decompensated cirrhosis, with the NRS-2002. The RFH-NPT was validated against the RFH-GA as an easy and quick assessment in a UK multicentre trial^(13,35)^, only taking about 3 min to finish. It takes into account objective symptoms and subjective feelings. It is based on fluid overload, weight loss and oral intake and eliminates the need assess muscle or fat loss and functional status. Moreover, the RFH-NPT also takes into consideration subclinical fluid retention. These findings contribute to RFH-NPT having a better ability to identify the risk of malnutrition.

One study found the RFH-NPT to be a useful predictor of disease progression and outcome in patients with chronic liver disease; the NRS-2002 was also found to be a successful predictor of mortality based on nutritional risk status in patients with liver cirrhosis^(16)^. Another study showed that the risk of malnutrition according to the RFH-NPT and not that assessed with other methods tended to be associated with mortality^(10)^. Our study shows that the RFH-NPT is an independent predictive factor for survival and is superior to the NRS-2002 for the prediction of survival based on nutritional risk in liver cirrhosis patients. Here, our results show that >28 % (7/28, online Supplementary Table S7) more of the non-surviving patients with decompensated cirrhosis were identified as being at risk of malnutrition by the RFH-NPT than by the NRS-2002.

Several other tools that are available to predict the risk of malnutrition were also assessed, such as the RFH-GA, MUST and LDUST. A previous study showed that MUST scores were relatively better correlated with the European Society for Clinical Nutrition and Metabolism criteria for the definition of malnutrition; however, the MUST is intended to be used as a general nutritional risk screening tool and is not specific to liver cirrhosis^(12,36)^. The LDUST is a liver disease-specific tool^(11)^. The LDUST consists of a series of six patient-directed questions covering the domains of nutrient intake, weight loss, subcutaneous fat loss, muscle mass loss, fluid accumulation and decline in functional status to determine whether undernutrition is present or absent. This tool may have limitations because it relies on the patient’s subjective judgement of each of the measured parameters. Preliminary data suggest that the LDUST has a high positive predictive but a low negative predictive value in patients with cirrhosis, leading to the conclusion that it is a negative screening tool is unable to reliably rule out undernutrition. When compared with the RFH-GA in our study, the LDUST had the highest sensitivity and lowest specificity (69 and 10 %, respectively), and the MUST had the lowest sensitivity (38·6 %). In addition, the present study showed that the RFH-NPT was better able than the LDUST to predict the risk of malnutrition associated with increased mortality^(17)^. Therefore, we did not consider the LDUST and MUST in the subsequent analyses, instead focusing on the NRS-2002, which is relatively more widely used in clinical practice in China, and the RFH-NPT. However, a higher quality controlled study should be conducted in a large population of liver cirrhosis patients to determine the best tool for the identification of nutritional risk.

The various conventional parameters, such as BMI, MAC and TSF, reflect malnutrition and the severity of liver disease to a certain extent^(37)^, but they are insufficient for the evaluation of the risk of malnutrition^(3,25,38)^. BMI has been found to be inaccurate in patients with ascites^(39,40)^. The MAC and TSF have variable results and have not been found to be strong predictors of malnutrition^(40,41)^. The serum level of albumin is a traditional marker that is thought to reflect liver synthetic function rather than nutritional status^(42)^, and the use of the level of prealbumin is controversial with regard to screening for patients at risk of malnutrition and predicting mortality^(43)^. It has been suggested that the TLC decreases with increasing levels of malnutrition and correlates with morbidity and mortality in hospitalised patients^(44)^. In our study, both the NRS-2002 and RFH-NPT were related to disease severity.

Nutritional status is associated with disease deterioration. The Child–Pugh class reflects the severity of liver disease. We investigated whether the RFH-NPT or the NRS-2002 was more sensitive for the identification of the risk of malnutrition risk at a relatively early stage of liver cirrhosis according to the Child–Pugh class. The RFH-NPT and the NRS-2002 were equivalent with regard to their ability to identify the risk of malnutrition in patients with Child–Pugh class A liver cirrhosis. The RFH-NPT identified a larger proportion of patients with Child–Pugh class B liver cirrhosis as being at risk for malnutrition than did the NRS-2002; however, the difference was NS, and the result needs to be confirmed in a larger sample of patients. However, the RFH-NPT identified 22 % (13/59) more patients with Child–Pugh class C disease who were at risk of malnutrition than did the NRS-2002. In practice, two simple criteria can be used immediately in patients at high risk of malnutrition, and one is having advanced decompensated cirrhosis (Child–Pugh class C). Patients with Child–Pugh class C disease are at very high risk of malnutrition; these patients do not need to be screened for malnutrition and instead can proceed directly to a nutritional assessment.

Here, we found that the RFH-NPT is better able to identify the risk of malnutrition (55/59 or 93·2 %) in patients with Child–Pugh class C disease, confirming the abovementioned proposition. The patients with Child–Pugh class B and C disease should receive nutritional interventions as soon as possible before any clinical sign of malnutrition is detectable, while patients with Child–Pugh class A disease should undergo more rigorous assessments to enable the provision of support in a timely manner^(34)^. Therefore, it is easy and quick for clinical staff to use the RFH-NPT to identify patient who are at risk of malnutrition instead of waiting for the evaluation of the Child–Pugh class before performing a detailed nutrition assessment.

Sarcopenia is a syndrome characterised by skeletal muscle loss that occurs with ageing, which is a major feature of malnutrition, particularly in patients with less severe hepatic dysfunction^(45)^. Previous studies have noted that the impact of sarcopenia was significant in patients with low MELD scores (<15) but not in patients with high MELD scores (≥15)^(46)^. Our results suggest that the RFH-NPT is more sensitive than the NRS-2002 for the identification of patients with a moderate to high risk of malnutrition among those with a MELD score <15.

Our study has limitations because it was performed with data from a single centre. First, we enrolled a small amount of patients in the evaluation of the sensitivity and specificity of the NRS-2002 and RFH-NPT, and therefore, more prospective studies are required. However, the incorporation of anthropometric and biochemical parameters reflecting different aspects of malnutrition increased our ability to differentiate between these tools. Second, some data were subjective assessments made by the patients and clinical staff, leading to potential recall bias and observer bias.

### Conclusion

This prospective study evaluated the efficacy of screening for nutritional risk with the RFH-NPT compared with the NRS-2002 in patients with cirrhosis primarily caused by hepatitis virus infection in China. The RFH-NPT was better able to predict the risk of malnutrition in patients with cirrhosis and had a superior prognostic value. Fewer patients at risk for malnutrition in the early stage of cirrhosis were misclassified by the RFH-NPT; therefore, the RFH-NPT can aid in identifying patients who need nutritional interventions in a timely manner to reduce complications. Future studies are needed to test the prognostic power of the RFH-NPT in larger populations.

## Supplementary material

For supplementary material accompanying this paper visit https://doi.org/10.1017/S0007114520002366. click here to view supplementary material
