# Supplementary material for: Royal Free Hospital-Nutritional Prioritizing Tool improves the prediction of malnutrition risk outcomes in liver cirrhosis patients compared with Nutritional Risk Screening 2002
Source: Br J Nutr. 2020 Dec 28;124(12):1293–302. doi: 10.1017/S0007114520002366 (PMC7656665; doi:10.1017/S0007114520002366)
Supplement: Supplementary file 1 [file S0007114520002366sup001.docx]

**Table S1 Overall characteristics in patients with compensated and decompensated cirrhosis**

| Index | Cirrhosis | |  | P |
| --- | --- | --- | --- | --- |
|  | Compensation | Decompensation |  |  |
| Age | 53.00±11.89 | 50.65±12.77 |  | 0.28 |
| BMI (kg/m^2^) | 24.52±3.90 | 23.24±3.66 |  | 0.051 |
| MAC (cm) | 28.81±3.36 | 26.42±3.62 |  | <0.001 |
| MAMC (cm) | 19.88±2.84 | 20.21±3.01 |  | 0.512 |
| TSF (mm) | 28.92±11.11 | 19.60±10.73 |  | <0.001 |
| Total serum protein (g/L) | 68.22±7.33 | 64.45±10.43 |  | 0.02 |
| Albumin (g/L) | 38.86±4.98 | 32.24±6.26 |  | <0.001 |
| Prealbumin (mg/L) | 164.81±61.19 | 90.95±58.19 |  | <0.001 |
| Total bilirubin (1 µmol/L) | 24.77±37.76 | 85.87±128.19 |  | 0.001 |
| Sodium(Na) (mmol/L) | 142.03±2.80 | 138.63±6.46 |  | 0.001 |
| Serum creatinine(SCr) (µmol/L) | 59.37±18.48 | 71.75±74.02 |  | 0.25 |
| Blood urea nitrogen(BUN) (mmol/L) | 4.72±1.87 | 6.02±4.70 |  | 0.06 |
| Ferroprotein (ng/mL) | 191.48±137.79 | 325.52±441.13 |  | 0.26 |
| International normalized ratio (INR) | 1.50±1.95 | 1.61±0.61 |  | 0.58 |
| Prothrombin time (PT) (s) | 14.89±2.76 | 18.66±5.60 |  | <0.001 |
| Plasma thromboplastin antecedent (PTA) (%) | 72.50±17.37 | 51.94±16.59 |  | <0.001 |
| Total lymphocyte count (×10^9^/L) | 1.43±0.94 | 0.97±0.63 |  | <0.001 |
| Blood ammonia (µmol/L) | 38.49±20.14 | 62.29±29.92 |  | 0.008 |
| Child-Pugh score | 6.41±1.34 | 9.58±2.28 |  | <0.001 |
| MELD score | 5.10±6.67 | 10.89±8.44 |  | <0.001 |
| **Sex** |  |  | Total |  |
| Male | 26(16.8%) | 68(43.9%) | 94(60.7%) | 0.19 |
| Female | 23(14.8%) | 38(24.5%) | 61(39.3%) |  |
| Total | 49(31.6%) | 106(68.4%) | 155(100%) |  |
| **NRS-2002** |  |  | Total |  |
| Low Risk | 38(24.5%) | 38(24.5%) | 76(49%) | <0.001 |
| Moderate to high risk | 11(7.1%) | 68(43.9%) | 79(51%) |  |
| Total | 49(31.6%) | 106(78.4%) | 155(100%) |  |
| **RFH-NPT** |  |  | Total |  |
| Low Risk | 38(24.5%) | 19(12.3%) | 57(36.8%) | <0.001 |
| Moderate to high risk | 11(7.1%) | 87(56.1%) | 98(63.2%) |  |
| Total | 49(31.6%) | 106(78.4%) | 155(100%) |  |
| **Child-Pugh class** |  |  | Total |  |
| A | 33(21.3%) | 11(7.1%) | 44(28.4%) | <0.001 |
| B | 13(8.4%) | 39(25.2%) | 52(33.5%) |  |
| C | 3(1.9%) | 56(36.1%) | 59(38.1%) |  |
| Total | 49(31.6%) | 106(68.4%) | 155(100%) |  |
| **MELD score** |  |  | Total |  |
| <15 | 46(29.7%) | 72(46.5%) | 118(76.1%) | <0.001 |
| >15 | 3(1.9%) | 34(21.9%) | 37(23.9%) |  |
| Total | 49(31.6%) | 106(68.4%) | 155(100%) |  |
| **Hepatic encephalopathy** |  |  | Total |  |
| Absent | 49 (31.6%) | 91(58.7%) | 140(90.3%) | 0.003 |
| Present | 0(0.0%) | 15(9.7%) | 15(9.7%) |  |
| Total | 49(31.6%) | 106(68.4%) | 155(100%) |  |
| **Ascites** |  |  | Total |  |
| Absent | 47(30.3%) | 36(23.2%) | 83(53.5%) | <0.001 |
| Present | 2(1.3%) | 70(45.2%) | 72(46.5) |  |
| Total | 49(31.6%) | 106(68.4%) | 155(100%) |  |

**Table S2 Patients’ characteristics in the low and high malnutrition risk group stratified by the NRS-2002 and RFH-NPT respectively.**

| Index | NRS-2002 | |  | P | RFH-NPT | |  | P |
| --- | --- | --- | --- | --- | --- | --- | --- | --- |
|  | Low Risk | High Risk |  |  | Low Risk | High Risk |  |  |
| Age | 54.18±11.71 | 48.71±12.74 |  | <0.01 | 53.11±10.91 | 50.40±13.30 |  | 0.20 |
| BMI (kg/m^2^) | 25.34±3.08 | 22.00±3.67 |  | <0.01 | 25.03±3.50 | 22.84±3.71 |  | <0.001 |
| MAC (cm) | 28.73±3.15 | 25.68±3.59 |  | <0.01 | 28.94±3.31 | 26.15±3.54 |  | <0.001 |
| MAMC (cm) | 20.23±2.70 | 20.00±3.19 |  | 0.626 | 20.21±2.90 | 20.04±3.00 |  | 0.706 |
| TSF (mm) | 27.16±12.02 | 18.08±9.41 |  | <0.01 | 28.00±11.75 | 19.36±10.41 |  | <0.001 |
| Total serum protein (g/L) | 66.30±8.42 | 65.01±10.79 |  | 0.41 | 68.17±7.76 | 64.17±10.41 |  | 0.013 |
| Albumin (g/L) | 35.14±7.34 | 33.56±5.81 |  | 0.14 | 37.91±5.47 | 32.26±6.38 |  | <0.001 |
| Prealbumin (mg/L) | 136.11±67.47 | 94.97±64.61 |  | 0.001 | 151.65±62.42 | 91.03±62.32 |  | <0.001 |
| Total bilirubin (µmol/L) | 35.25±53.78 | 96.67±141.24 |  | 0.001 | 35.57±68.29 | 85.73±126.81 |  | 0.005 |
| Sodium(Na) (mmol/L) | 140.58±4.58 | 138.87±6.65 |  | 0.07 | 141.60±3.33 | 138.61±6.58 |  | 0.002 |
| Serum creatinine(SCr) (µmol/L) | 63.22±35.02 | 72.27±80.20 |  | 0.37 | 57.53±14.03 | 73.83±77.07 |  | 0.12 |
| Blood urea nitrogen(BUN) (mmol/L) | 5.19±2.17 | 6.01±5.27 |  | 0.21 | 4.60±1.29 | 6.20±4.93 |  | 0.228 |
| Ferroprotein (ng/mL) | 230.36±199.67 | 310.41±446.84 |  | 0.42 | 134.08±104.26 | 341.46±422.42 |  | 0.09 |
| International normalized ratio (INR) | 1.33±0.26 | 1.81±1.63 |  | 0.01 | 1.28±0.27 | 1.75±1.47 |  | 0.02 |
| Prothrombin time (PT) (s) | 16.25±2.53 | 18.64±6.64 |  | 0.004 | 15.76±2.50 | 18.46±6.03 |  | 0.002 |
| Plasma thromboplastin antecedent PTA (%) | 63.78±17.33 | 53.21±20.04 |  | 0.002 | 67.47±18.71 | 52.56±17.48 |  | <0.001 |
| Total lymphocyte count (10^9^/L) | 1.20±0.88 | 1.03±0.62 |  | 0.19 | 1.32±0.96 | 1.00±0.60 |  | 0.01 |
| Blood ammonia (µmol/L) | 54.51±25.43 | 60.00±32.50 |  | 0.46 | 53.08±26.17 | 59.24±30.89 |  | 0.46 |
| Child-Pugh score | 7.57±2.21 | 9.54±2.39 |  | <0.01 | 6.67±1.59 | 9.68±2.26 |  | <0.001 |
| MELD score | 6.41±5.99 | 11.6±9.47 |  | <0.01 | 5.25±5.62 | 11.28±8.89 |  | <0.001 |
|  |  |  |  |  |  |  |  |  |
| Sex |  |  | Total |  |  |  | Total |  |
| Male | 47(30.3%) | 47(30.3%) | 94(60.6%) | 0.77 | 33(21.3%) | 61(39.3%) | 94(60.6%) | 0.59 |
| Female | 29(18.7%) | 32(20.7%) | 61(39.4%) |  | 24(15.5%) | 37(23.9%) | 61(39.4%) |  |
| Total | 76(49.0%) | 79(51%) | 155(100%) |  | 57(36.8%) | 98(63.2%) | 155(100%) |  |
| Cirrhosis |  |  | Total |  |  |  | Total |  |
| Compensated | 38(24.5%) | 11(7.1%) | 49(31.6%) | <0.001 | 38(24.5%) | 11(7.1%) | 49(31.6%) | <0.001 |
| Decompensated | 38(24.5%) | 68(43.9%) | 106(68.4%) |  | 19(12.3%) | 87(56.1%) | 106(68.4%) |  |
| Total | 76(49.0%) | 79(51%) | 155(100%) |  | 57(36.8%) | 98(63.2%) | 155(100%) |  |
| Child-Pugh class |  |  | Total |  |  |  | Total |  |
| A | 34(21.9%) | 10(6.5%) | 44(28.4%) | <0.001 | 38(24.5%) | 6(3.9%) | 44(28.4%) | <0.001 |
| B | 25(16.1%) | 27(17.4%) | 52(33.5%) |  | 18(11.6%) | 34(21.9%) | 32(33.5%) |  |
| C | 17(11.0%) | 42(27.1%) | 59(38.1%) |  | 4(2.6%) | 55(35.5%) | 59(38.1) |  |
| Total | 76(49.0%) | 79(51%) | 155(100%) |  | 57(36.8%) | 98(63.2%) | 155(100%) |  |
| MELD score |  |  | Total |  |  |  | Total |  |
| <15 | 66(42.6%) | 52(33.5%) | 118(76.1%) | 0.002 | 54(34.8%) | 64(41.3%) | 118(76.1%) | <0.001 |
| >15 | 10(6.5%) | 27(17.4%) | 37(23.9%) |  | 3(1.9%) | 34(21.9%) | 37(23.9%) |  |
| Total | 76(49.0%) | 79(51%) | 155(100%) |  | 57(36.8%) | 98(63.2%) | 155(100%) |  |
| Hepatic encephalopathy |  |  | Total |  |  |  | Total |  |
| Absent | 74(47.7%) | 66(42.6%) | 140(90.3%) | 0.008 | 57(36.8%) | 26(16.8%) | 83(53.5%) | <0.001 |
| Present | 2(1.3%) | 13(8.4%) | 15(9.7%) |  | 0(0.%) | 72(46.5%) | 72(46.5%) |  |
| Total | 76(49.0%) | 79(51%) | 155(100%) |  | 57(36.8%) | 98(63.2%) | 155(100%) |  |
| Ascites |  |  | Total |  |  |  | Total |  |
| Absent | 56(36.1%) | 27(17.4%) | 83(53.5%) | <0.001 | 56(36.1%) | 84(54.2%) | 140(90.3%) | 0.024 |
| Present | 20(12.9%) | 52(33.5%) | 72(36.5%) |  | 1(0.6%) | 14(9.1%) | 15(9.7%) |  |
| Total | 76(49.0%) | 79(51%) | 155(100%) |  | 57(36.8%) | 98(63.2%) | 155(100%) |  |

**Table S3 Nutrition risk screening comparison between the NRS-2002 and RFH-NPT in patients with compensated cirrhosis**

| NRS-2002 | RFH-NPT | | Total | P |
| --- | --- | --- | --- | --- |
|  | Low risk | High risk |  |  |
| Low risk | 36 | 2 | 38 |  |
| High risk | 2 | 9 | 11 | 1.00 |
| Total | 38 | 11 | 49 |  |

**Table S4 Nutrition risk screening comparison between the NRS-2002 and RFH-NPT in patients with decompensated cirrhosis**

| NRS-2002 | RFH-NPT | | Total | P |
| --- | --- | --- | --- | --- |
|  | Low risk | High risk |  |  |
| Low risk | 15 | 23 | 38 |  |
| High risk | 4 | 64 | 68 | <0.001 |
| Total | 19 | 89 | 106 |  |

**Table S5 Nutrition risk screening comparison between the NRS-2002 and RFH-NPT in patients with or without ascites.**

| Ascites | RFH-NPT | NRS-2002 | | Total | P |
| --- | --- | --- | --- | --- | --- |
|  |  | Low risk | High risk |  |  |
| Absent | Low risk | 51 | 6 | 57 | 1 |
|  | High risk | 5 | 21 | 26 |  |
|  | Total | 56 | 27 | 83 |  |
| Present | Low risk | 0 | 0 | 0 | - |
|  | High risk | 20 | 52 | 72 |  |
|  | Total | 20 | 52 | 72 |  |

**Table S6 Nutrition risk screening comparison between the NRS-2002 and RFH-NPT in patients with or without hepatic encephalopathy.**

| Hepatic encephalopathy | RFH-NPT | NRS-2002 | | Total | P |
| --- | --- | --- | --- | --- | --- |
|  |  | Low risk | High risk |  |  |
| Absent | Low risk | 50 | 6 | 56 | 0.001 |
|  | High risk | 24 | 60 | 84 |  |
|  | Total | 74 | 66 | 140 |  |
| Present | Low risk | 1 | 0 | 1 | 1 |
|  | High risk | 1 | 13 | 14 |  |
|  | Total | 2 | 13 | 15 |  |

**Table S7 Nutrition risk screening comparison between the NRS-2002 and RFH-NPT in survival patients.**

| Status | Cirrhosis | RFH-NPT | NRS-2002 | | Total | P |
| --- | --- | --- | --- | --- | --- | --- |
|  |  |  | Low risk | High risk |  |  |
| Living | Compensated | Low risk | 31 | 2 | 33 | 1 |
|  |  | High risk | 2 | 9 | 11 |  |
|  |  | Total | 33 | 11 | 44 |  |
|  | Decompensated | Low risk | 11 | 4 | 15 | 0.031 |
|  |  | High risk | 14 | 41 | 55 |  |
|  |  | Total | 25 | 45 | 70 |  |
|  | Overall | Low risk | 42 | 6 | 48 | 0.052 |
|  |  | High risk | 16 | 50 | 66 |  |
|  |  | Total | 58 | 56 | 114 |  |

**Table S8 Nutrition risk screening comparison between the NRS-2002 and RFH-NPT in deceased patients.**

| Status | Cirrhosis | RFH-NPT | NRS-2002 | | Total | P |
| --- | --- | --- | --- | --- | --- | --- |
|  |  |  | Low risk | High risk |  |  |
| Deceased | Compensated | Low risk | - | - | - |  |
|  |  | High risk | - | - | - |  |
|  |  | Total | - | - | - |  |
|  | Decompensated | Low risk | 4 | 0 | 4 | 0.016 |
|  |  | High risk | 7 | 14 | 21 |  |
|  |  | Total | 11 | 14 | 25 |  |
